# The relationship between reflex eye realignment and the percept of single vision in young children

**DOI:** 10.1038/s41598-020-78636-0

**Published:** 2021-01-11

**Authors:** Kimberly Meier, Deanna L. Lundell, Eric S. Seemiller, Deborah Giaschi, Laurie M. Wilcox, T. Rowan Candy

**Affiliations:** 1 Department of Psychology, University of Washington, 119A Guthrie Hall Box 351525, Seattle, WA 98195 USA; 2 School of Optometry and Vision Science, Indiana University, 800 E Atwater Avenue, Bloomington, IN 47405 USA; 3 Department of Ophthalmology and Visual Sciences, University of British Columbia, 4480 Oak Street, Vancouver, BC V6H 3V4 Canada; 4 Department of Psychology, Centre for Vision Research, York University, 4700 Keele St, Toronto, ON M3J 1P3 Canada; 5 Programs in Neuroscience and Cognitive Science, Indiana University, Bloomington, USA

**Keywords:** Visual system, Oculomotor system, Sensorimotor processing

## Abstract

Effective binocular vision is dependent on both motor and perceptual function. Young children undergo development of both components while interacting with their dynamic three-dimensional environment. When this development fails, eye misalignment and double vision may result. We compared the range of image disparities over which young children display reflex motor realignment of their eyes with the range over which they report a single versus double percept. In response to step changes in the disparity of a 2.2° wide stimulus, 5-year-olds generated an adult-like reflex vergence velocity tuning function peaking at 2° of disparity, with a mean latency of 210 ms. On average, they reported double vision for stimulus disparities of 3° and larger, compared to 1° in adult reports. Three-year-olds also generated reflex vergence tuning functions peaking at approximately 2° of disparity, but their percepts could not be assessed. These data suggest that, by age 5, reflex eye realignment responses and percepts driven by these brief stimuli are tightly coordinated in space and time to permit robust binocular function around the point of fixation. Importantly, the plastic neural processes maintaining this tight coordination during growth control the stability of visual information driving learning during childhood.

## Introduction

For typical visual development to occur, infants and young children must coordinate the alignment of their two eyes with the neural integration of sensory information from the eyes to achieve binocular vision in a dynamic three-dimensional environment^1,2^. This coordination is apparently disrupted when young children develop manifest eye misalignment, strabismus, during the first years after birth^3^. Among others, Banks et al.^4^ have demonstrated the importance of aligned binocular visual experience during the first years after birth. However, while numerous studies have documented the development of either motor or sensory binocular performance, the relationship between motor eye alignment and perceptual integration of retinal images in maintaining fused binocular vision for young children is largely unknown (although see^1,4–6^). The current study forms a first step towards characterizing the dynamic relationship between the motor and perceptual visual systems in achieving reflex fusion of misaligned targets during early childhood, a time when children are learning about their dynamic three-dimensional environment, are calibrating their responses during rapid growth, and are at highest risk for permanent disruption of binocular vision. This relationship between motor and perceptual systems defines the stability of information available to later stages of the brain during early learning and provides clinicians with a reference for typical function that cannot be gained currently in the clinic.

What might this relationship be during early childhood? If the brain is to integrate information falling on corresponding points in the two retinal images to form a fused percept, the fovea of each eye must be reliably aligned at or near the desired point of fixation so that the images correspond. This motor vergence alignment response is, however, driven by information derived from the two retinal images. The simplest hypothesis might be that the presence of a target in a different depth plane results in the perception of misaligned retinal images, which drives a vergence alignment response and then the perception of a fused binocular target. As adults our typical perception, however, is of a unified stable world without an awareness of misaligned images undergoing motor alignment prior to perceptual fusion, as we look between objects on a desk for example. There is a region of space in front of and behind the fixation plane where points with binocular disparity appear fused (Panum’s fusional area), providing the percept of single vision^7^. Do children also experience a unified stable world, or are they more likely to perceive image misalignment and double vision while they undertake their vergence motor responses? What range of retinal disparities is capable of driving a reflex vergence response and therefore providing robust binocular motor behavior? How stable are fused percepts during these responses? Could an immaturity in the motor-perception relationship be a factor in the development of manifest misalignment and strabismus?

It could be that (i) young children have a protective perceptual mechanism relative to adults that supports single vision despite immature inaccuracies in vergence that would result in diplopia for adults (as suggested by Aslin^8^ and Giaschi et al.^9^). At the opposite end of a continuum of relationships, it could be that (ii) the reflex vergence system responds to a wider range of disparities during early childhood than in adults to enable robust motor alignment while the perceptual processes mature to eliminate diplopia. In starting to address these questions, we introduced brief step changes in retinal disparity to determine both the range of disparities over which young children experience a single percept prior to their vergence response being completed, and the range of disparities capable of driving reflex vergence in these children.

Vergence eye alignment responses have been recorded from human neonates within days after birth^8,10,11^, and typical infants can make vergence tracking responses to less than 2° of full-cue movement in depth^12^ or oscillation of disparity alone in large visual stimuli^13^ by two months of age. At three to five months of age, they are typically also able to make incremental reflex fusional vergence responses up to a total of 10–15 prism diopters (6°–9°)^14^. Although the percepts of infants are not directly accessible, behavioral and EEG/VEP responses to binocular correlograms have been recorded from a small proportion of infants by two months of age^15–18^ (reviewed in^19^), and both behavioral and EEG responses to relative disparity against a background can be generated by most infants by three to six months of age (reviewed in^20^). These studies indicate that binocular function undergoes rapid development during the first months after birth.

As typical infants grow to the ages at which strabismus most commonly develops (typically prior to age 6 years^3^) visual demands are changing due to physical growth. Children must recalibrate their motor alignment responses with the increasing distance between their eyes while apparently maintaining eye alignment and perceptual fusion during their habitual activities. At 4 to 5 years of age, children become able to complete psychophysical tasks that require verbal report. Interestingly, reports of whether misaligned targets were perceived as single/fused or double/diplopic indicated that 4- to 5-year old children had a fusional area over half a degree larger than adults^9^. This is consistent with a proposal that infants might have a larger fusional area prior to the fine-tuning of accurate binocular eye alignment^8^, providing a protective mechanism against diplopia into the early school-age years.

In this study, children of 3–4 and 5–6 years of age were presented with brief jumps in retinal disparity of small fixated targets while their vergence motor alignment responses were recorded. The 5–6-year-old children went on to report whether targets were perceived as fused or diplopic during repeat presentation of the same stimuli. The results indicate that reflex vergence responses are well-coordinated with reports of single vision by 5 years, and that 3-year-olds demonstrate similar reflex vergence responses to these briefly presented small disparities. The tuning of these responses to a limited range of disparity indicates the tolerance of reflex vergence and perceptual fusion responses to unstable alignment at these important preschool ages when strabismus and amblyopia can lead to permanent disruption of vision.

## Results

### Perceptual task

**Figure 1:**
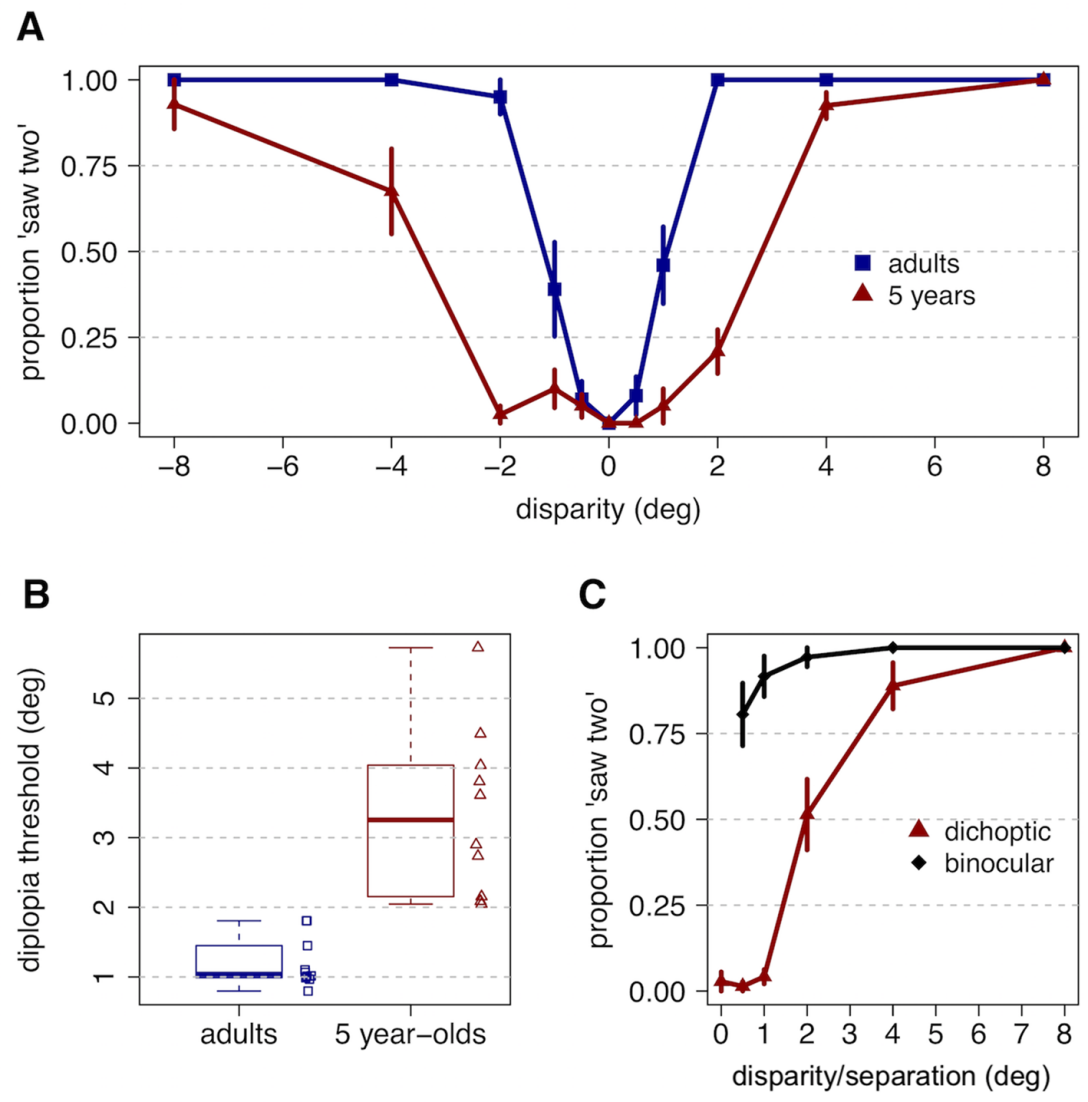
(**A**) Mean proportion of trials for which participants reported seeing two (rather than one) characters as a function of disparity, for adults (n = 10) and 5-year-olds (n = 10). Error bars reflect standard error across participants. (**B**) The median and distribution of participants’ 63% diplopia thresholds, collapsed across sign, for each age group. Symbols to the right represent individual participant estimates. C: Mean proportion of stimuli reported as ‘saw two’ by the group of 5-year-olds who took part in the interleaved binocular control condition, collapsed across disparity sign. Error bars reflect standard error across participants.

Figure 1 displays the mean proportion of stimuli reported as ‘saw two’, rather than one, by participants in each age group as a function of the size of the disparity step (panel A), and the distributions of diplopia thresholds across participants for each age group (panel B). To calculate each participant’s 63% diplopia threshold, their proportions of ‘saw two’ responses were first collapsed across disparity sign. The median thresholds were 1.0° (*SD* = 0.4) and 3.3° (*SD* = 1.2) for adults and 5-year-olds, respectively. The children had a significantly greater median diplopia threshold (Kruskal–Wallis χ^2^ (1) = 14.30, *p* < 0.001; *η*^2^ = 0.74) than the adults, suggesting that 5-year-olds have a larger perceptual fusional area than adults for these stimuli. Moreover, while adult diplopia thresholds were not significantly different as a function of sign (paired Wilcoxon signed rank test *V* = 26, *p* = 0.29), the median diplopia threshold for 5-year-olds’ responses to nearer stimuli was significantly larger than for more distant stimuli (2.9 vs. 2.5°; *V* = 36, *p* = 0.014). While diplopia thresholds for near were not correlated with thresholds for the distant stimuli in adults, *r*(8) = 0.35, 95% CI [− 0.36, 0.80], *p* = 0.32, they were significantly correlated in children, *r*(8) = 0.83, 95% CI [0.38, 0.96], *p* = 0.0052, such that children with larger diplopia thresholds for the near direction also had larger thresholds in the distant direction. Finally, the diplopia thresholds assessed in children were significantly more variable than those in adults, *F*(9,9) = 11.26*,* 95% CI [2.79, 45.32], *p* = 0.001. The variability in children’s diplopia threshold was not related to mean vergence latency (*r*(8) = − 0.04, 95% CI [− 0.65, 0.61], *p* = 0.92) or velocity (*r*(8) =  − 0.02, 95% CI [− 0.64, 0.62], *p* = 0.96) on the vergence task described below.

Results from the control condition, in which identical doubled binocular images were interleaved (Fig. 5), confirmed that 5-year-old children understood the requirements of the task (Fig. 1, panel C). Under binocular rather than dichoptic presentation, children reported seeing two characters at small separations where the characters overlapped. This suggests that the children were in fact perceiving a single fused character when they reported a single character under dichoptic presentation of the same disparities.

### Vergence task

**Figure 2:**
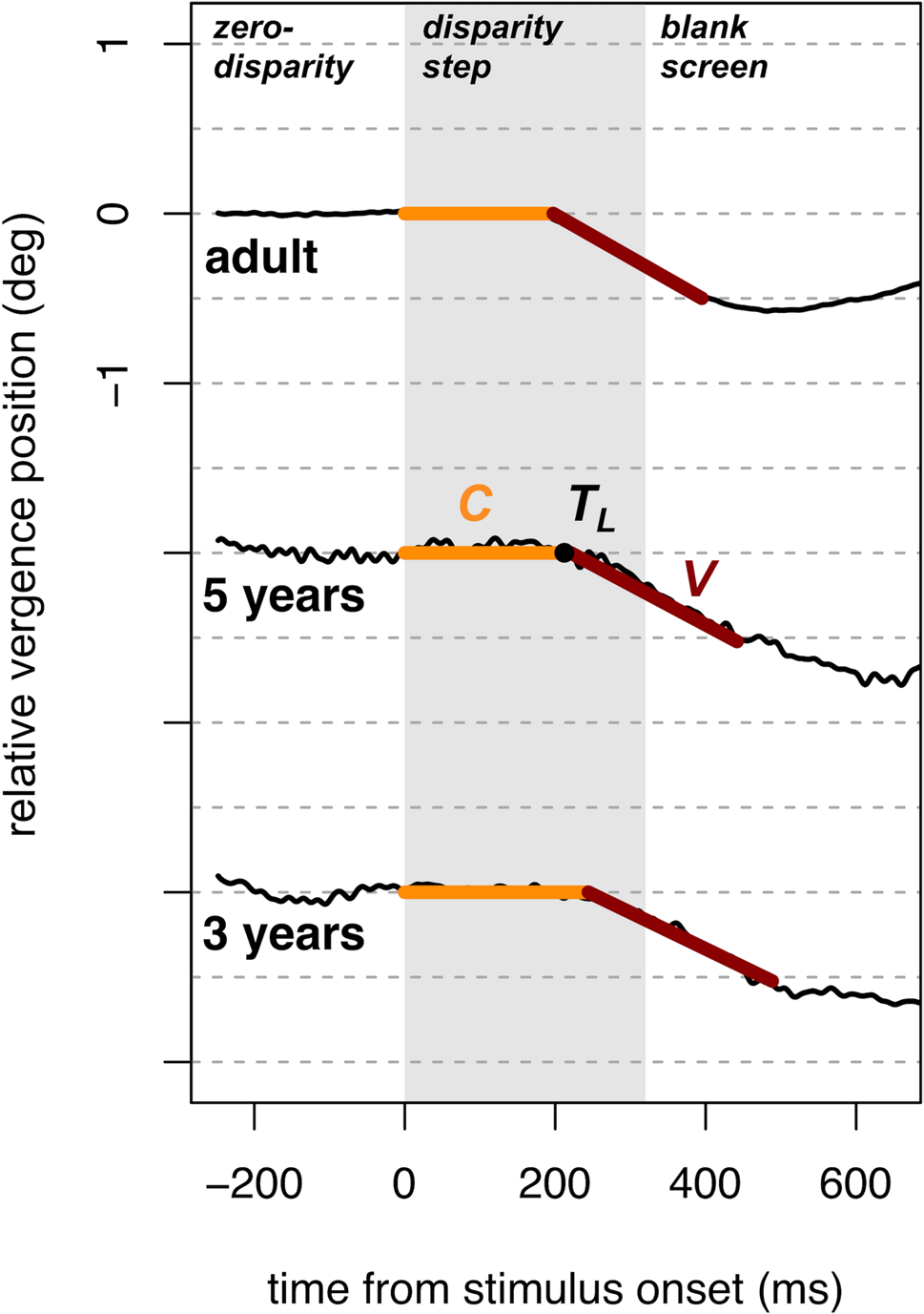
Representative averaged vergence responses collected from one participant in each age group, for stimuli stepped to − 2° disparity (negative disparity indicates a stimulus for convergence). The responses represent the mean vergence position across all trials completed at the relevant disparity (20 trials for the adult and 5-year-old; 10 trials for the 3-year-old). Parameters estimated from the bilinear function are labeled in the middle trace, where *C* is the stable vergence value for the first zero-disparity period, *T*_*L*_ is the latency to the vergence response, and *V* is the slope representing the summary velocity of the open-loop vergence response.

Representative averaged vergence responses to stimuli stepped to − 2° disparity for one participant from each age group are displayed in Fig. 2. These reflect the mean vergence position across all trials completed by each participant.

#### Latency to the vergence response

**Figure 3:**
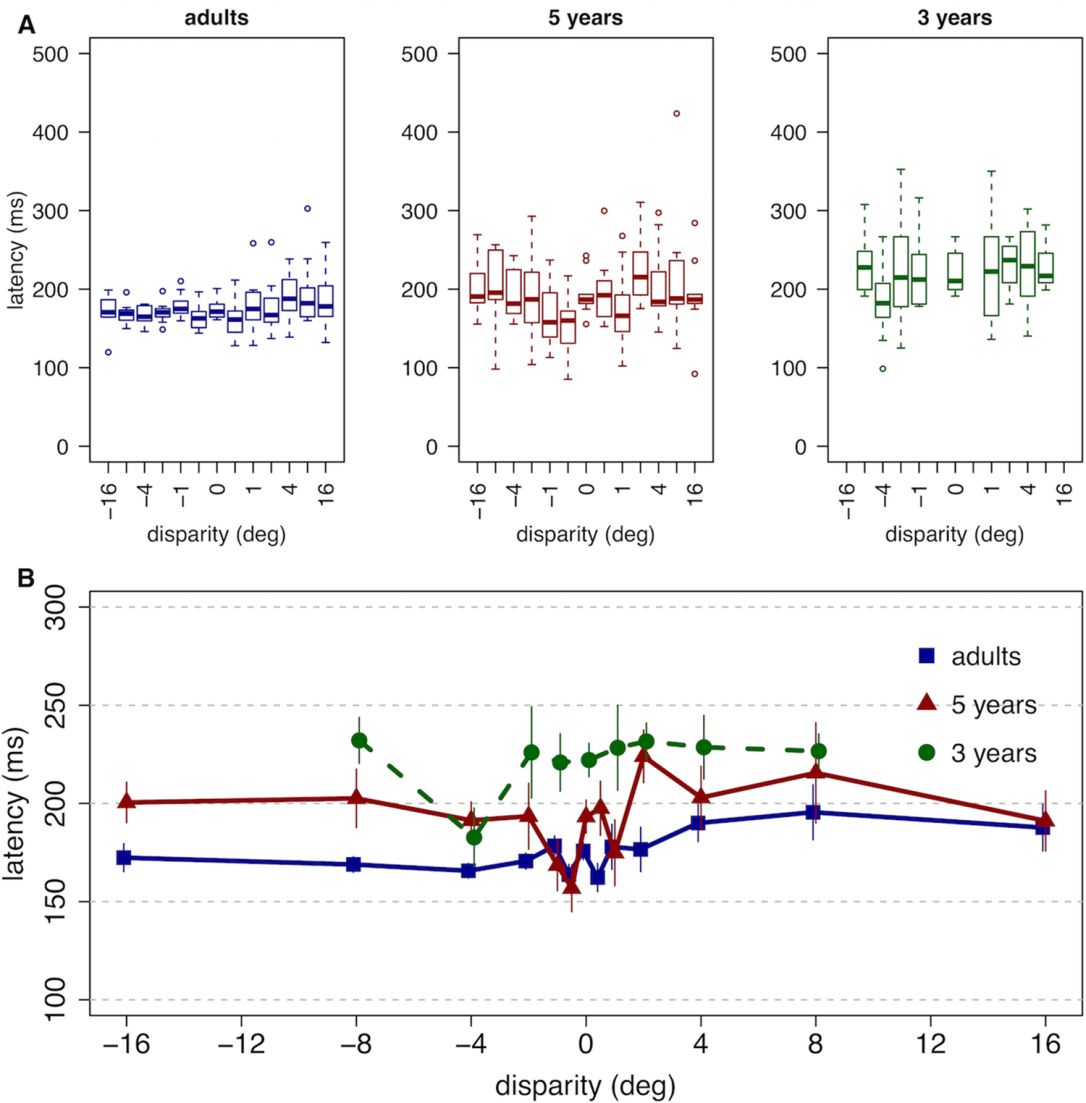
The latency between the step in stimulus disparity and initiation of the vergence response (T_L_). (**A**) The distributions of individual participant latencies, with median values, as a function of disparity step size for each age group (n = 10 per group). (**B**) Mean latency as a function of disparity step size for each group. Error bars reflect standard error. In all plots, the value plotted at a disparity of 0 reflects the mean latency across all trials, since no vergence movement is elicited from these stimuli.

The mean latency (T_L_), across participants and disparities, between the step in stimulus disparity and the beginning of the vergence movement was 183 ms for adults, 95% CI [175, 193]; 210 ms for the 5-year-olds, 95% CI [199, 221]; and 227 ms for the 3-year-olds, 95% CI [213, 241]. These distributions are shown in Fig. 3. A linear mixed model with age, disparity magnitude, disparity sign, and a disparity magnitude by sign interaction as fixed effects and participant as a random effect was fit to these data, with latency as the dependent variable. The parameters of the fits are shown in Table 1. The main effect of categorical age was significant, such that 5-year-olds and 3-year-olds had significantly longer latencies than adults. The main effects of continuous disparity magnitude and of disparity sign were not significant. There was a significant disparity by sign interaction such that the change in latency as a function of disparity magnitude was 3.3 ms/° steeper (longer) for stimuli presented at positive (uncrossed) disparities, which initiated divergent eye movements, than for stimuli presented at negative (crossed) disparities, which initiated convergence. Note that zero-disparity stimulus trials were not included in these analyses as no vergence movement is elicited from zero-disparity stimuli.

**Table 1:** Latency to the vergence response (T_L_) was fit with a linear mixed model including age (categorical), disparity magnitude (continuous), disparity sign, and a magnitude by sign interaction as fixed variables and participant as a random variable. Disparity sign is coded such that a positive beta indicates a longer latency (slower response) for stimuli presented at positive (far) disparities than those presented at negative (near) disparities.

Source	β (ms)	SE β	Z	*p*
Baseline (constant) (Adult)	163.9	10.6	15.5	< 0.001
Age: 5 years	26.6	12.8	2.07	0.038
Age: 3 years	47.4	13.3	3.55	< 0.001
Disparity magnitude	1.3 (ms/deg)	0.8	1.42	0.154
Disparity sign	8.9	8.6	1.03	0.301
Disparity magnitude × sign	3.3 (ms/deg)	1.2	2.65	0.008

#### Vergence response velocity

**Figure 4:**
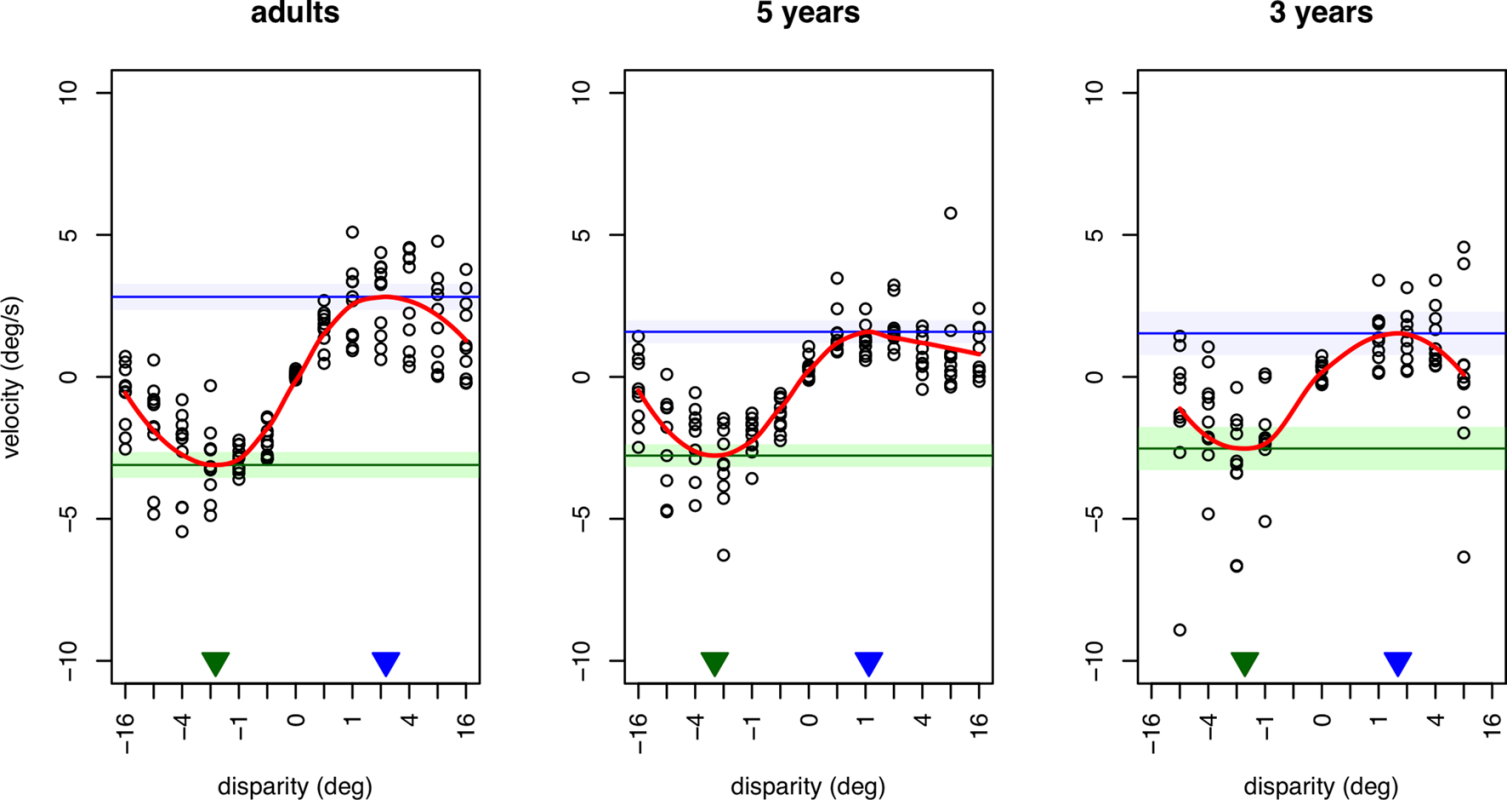
The velocity estimates from the averaged vergence responses collected from each participant in each age group (open circles; n = 10 per group). The curves fit to these data are LOESS functions. The horizontal green (lower) and blue (upper) lines indicate the peaks for the convergent and divergent responses, respectively, with the 95% confidence interval for these peak estimates (shaded region). Triangles indicate the stimulus disparity at these peak estimates.

The estimates of summary response velocity over a period equivalent to the latency (Fig. 2) for each participant at each disparity step size are shown in Fig. 4. A LOESS function^21^ was fit to each age group’s estimates as a function of disparity step size. This least-squares fit was performed with a smoothing parameter of 0.60. The minimum and maximum velocities from these fits were used to define the disparities at which the peak responses to near and far stimuli occurred for each age group, with the standard errors from the fits used to determine confidence intervals. The peak summary velocity (V) for the convergent responses was 3.1°/s, 95% CI [2.7, 3.6] for adults; 2.8°/s, 95% CI [2.4, 3.2] for 5-year-olds; and 2.5°/s, 95% CI [1.8, 3.3] for 3-year-olds. The peak divergent velocity was 2.8°/s, 95% CI [2.4, 3.3] for adults; 1.6°/s, 95% CI [1.2, 2.0] for 5-year-olds; and 1.5°/s, 95% CI [0.8, 2.5] for 3-year-olds. Within adults, peak summary velocity for near and far vergence eye movements were not significantly different (*M* difference = 0.3°/s, 95% CI [0.38, 0.92]). However, 5-year olds (*M* difference = 1.2°/s, 95% CI [0.69, 1.75] and 3-year-olds (*M* difference = 1.0°/s, 95% CI [0.11, 1.92]) showed significantly larger peak velocities in the negative/convergent direction than in the positive/divergent direction. These peaks were elicited from stimuli of approximately 2° disparity in each direction for all age groups.

## Discussion

The typically-developing visual system must coordinate bifoveal fixation and binocular single perception at the point of fixation to establish typical binocular vision (as discussed by^1^). In the natural dynamic three-dimensional environment, stable unified perception during reflex realignment of the eyes with small shifts in viewing distance is a fundamental component of robust binocular vision. This is not a trivial accomplishment in that it requires coordination of motor and perceptual processes on a millisecond timeframe^22^. In the absence of this coordination, the developing visual system can decompensate into strabismus, amblyopia and/or permanent loss of binocular function. In attempting to understand how both typical and atypical states might develop, the goal of this study was to examine the relationship between rapid reflex vergence responses and perception in establishing stable binocular vision for typically-developing young children.

To compare perceptual ranges of single vision for 5-year-old children and adults, participants were asked to report whether they saw one or two characters during brief step changes in disparity, before the eyes were able to make a motor realignment response. Comparison of the perceptual data with the binocular control condition (Fig. 1C) indicates that the 5-year-old children were capable of performing reliable psychophysics. A report of seeing two characters indicates simultaneous perception of misaligned characters, while a report of seeing one character indicates either perceptual fusion or suppression of one of the images. In either case, the data collected here suggest that the 5-year-old children maintained a stable single percept over steps of at least 1–2° from the plane of fixation during the short duration disparity steps (Fig. 1A). With the fixation plane at 62 cm, the 5-year-old 63% diplopia threshold range of approximately ± 3° corresponds to viewing distances from approximately 40–160 cm, whereas the adult range of approximately ± 1° corresponds to approximately 50–75 cm. Thus, these children appear capable of maintaining a single percept over a significant range of viewing distances while their vergence response is initiated. In support of the first hypothesis proposed in the introduction, we found the 5-year-old children had a larger fusional area than adults, a result that replicates Giaschi et al.^9^. While our data also indicate that children have a larger fusional area in the near/crossed direction, we caution that the total number of trials included in this calculation is low, so replication of this result is warranted before drawing strong conclusions about a direction asymmetry.

In the current study the vergence response to disparity was only assessed during a limited open-loop timeframe (a 320 ms step presentation) due to the potential for visual feedback after the realignment response was initiated. This feedback could indicate that the best image focus was still in the plane of the screen or that the size of the target had not changed with disparity, for example, and lead to disruption of the naturalistic vergence response in the presence of competing cues^23,24^. The vergence response data suggest that three-and 5-year-old children, on average, can initiate a reflex vergence response with a latency not more than 50 ms longer than that of adults, and therefore that this aspect of binocular function is not dramatically immature at these ages (Fig. 1B). The adult latencies of 150–200 ms are consistent with those reported routinely in previous studies of small foveal targets^25–31^. Few studies have reported on latency to pure vergence responses in 3- and 5-year old children, but the latencies in our children are notably shorter than the 300 – 500 ms previously reported in children aged 4–7 years^32^. That study used 16° disparity steps to LED targets, however, rather than the spatially broadband stimuli used here. The shape of the young participants’ velocity tuning functions (Fig. 4) were also remarkably similar to those of adults, with an approximately linear relationship between magnitude of disparity and vergence velocity out to a disparity step of approximately two° in either direction^33,34^. These responses are typically described using metrics of velocity because the velocity profile of a response has been demonstrated to resemble the firing patterns of the motor neurons controlling the response^35^. The linear relationship over the 2° range is also consistent with the concept of the main sequence relationship between response velocity and stimulus amplitude found in adults^30^. That the reflex vergence system responds to the same range of disparities during early childhood argues against the second hypothesis proposed in the introduction.

Thus, overall, there was good correspondence between the range of disparities over which the percept remained single and the reflex vergence system appeared responsive in 5-year-olds and adults, with the 5-year-olds' data suggesting that the immature perceptual range may extend further beyond the linear vergence range during development. This relationship supports hypothesis (i) that a perceptual mechanism is protective against diplopia during early childhood, and suggests that perception of double images is not required to drive a vergence movement by that age. Although of interest, it was not possible to assess the direct relationship between the vergence response and the perceptual report in individual trials in the current study as the two sets of data were collected in different sessions. It was also not possible to collect perceptual reports from the 3-year-old children unfortunately and, while their reflex motor data appear impressively adult-like, the extent to which their percepts might provide additional protection against diplopia is unclear. What is clear, however, is that they do not make fusional vergence responses to a significantly extended range of disparities.

It was not possible to evaluate the typical accuracy of the vergence responses at their endpoint as a result of the brief presentations and concern about conflicting cues, such as blur and size, in the stimuli. However, the velocity data do suggest a robust region of binocular function for this form of local stimulus around the point of fixation. The stability of perceptual fusion under these conditions has been documented in adults. As noted by Westheimer and Mitchell^36^, observers rarely report loss of fusion or the percept of diplopia during changes in vergence. For example, using targets only 11 arcmin wide, observers maintained a fused percept in the presence of up to 2° of overconvergence. In our case, the targets were much larger (2°) but the percept of fusion was maintained over a similar range of convergence demand. Similar to saccadic suppression^37^, *vergence suppression* could help maintain a stable percept of the world by reducing sensitivity to visual information during the execution of an eye movement. During the vergence movements of adults, suppression occurs from 200 ms prior to initiation until approximately 50–350 ms after^38–42^, with strongest suppression occurring at vergence onset. Though the development of vergence suppression has not been studied, vergence and saccadic suppression likely originate from the same central mechanism^40,42^, and children 12–14 years of age display stronger saccadic suppression than young adults^43^. The role of vergence suppression in the current study is not clear in that the participants were able to make consistent reports of their percepts of the targets after the 320 ms presentation, which is not consistent with suppression of perception for both eyes.

All three age groups generated slower velocity responses in the divergent than convergent direction, both in terms of latency and response velocity, although the velocity difference only reached statistical significance in the children. The velocity data are consistent with the previous adult literature^44^, even though the current data are presented in terms of a single summary response velocity rather than a response velocity profile (e.g.^22^). Of note, depending on a subject’s inter-pupillary distance, divergent stimuli of 5° or greater correspond to the unnatural situation of diverging the eyes beyond parallel alignment. At the larger disparities some of the individuals appeared to generate a response in the wrong direction (Fig. 4), i.e., a divergent response to crossed disparities or a convergent response to uncrossed disparities. This could reflect noise in the measurement and velocity estimate, or perhaps indicate movement to an idiosyncratic position (such as the participant’s latent heterophoria, a small misalignment only revealed in dissociated conditions^34^).

In summary, this study suggests that a coordinated relationship between reflex alignment of the eyes and the percept of a single target is in place by 5 years of age, and that 3-year-olds exhibit largely similar reflex vergence responses for these stimuli. Such capabilities are central to establishing stable robust binocular vision in a dynamic three-dimensional environment. This relationship defines the information available for experience-dependent visual learning and the maturation of processing in higher-level regions of the brain dependent on these low-level signals. The impact of the relationship and conditions such as anisometropia, which results in a mismatch in retinal image quality between the eyes, in the development of strabismus and amblyopia are to be determined. This study and Giaschi et al.^9^ suggest that perceptual fusion might be achieved over a somewhat larger area during childhood than found in adults, but that diplopia is likely to occur at most strabismic deviation angles during childhood. The operating ranges of these motor and perceptual responses are also relevant in the design of binocular content for new forms of augmented and virtual reality technologies proposed for education and therapeutic purposes during childhood (e.g.,^45–47^).

## Methods

The study was approved by the Indiana University Institutional Review Board and the University of British Columbia Behavioural Research Ethics Board. It was carried out in accordance with the Code of Ethics of the World Medical Association (Declaration of Helsinki). Health Insurance Portability and Accountability Act (HIPAA) authorization was obtained before participation and informed consent was gathered from all adult participants and guardians of the children. Verbal assent was gathered from all of the children.

### Participants

Thirteen children aged between 3.1 and 3.9 years (mean age = 3.6 years, *SD* = 0.32), 11 children aged between 5.2 and 6.2 years (mean age = 5.5 years, *SD* = 0.34), and 10 pre-presbyopic adults were recruited from the local community in Bloomington, Indiana. Two of these children and seven additional children aged between 5.4 and 6.8 years (mean age = 6.0 years, *SD* = 0.51) were recruited to participate in a control condition: four in Vancouver, BC, Canada and three in Bloomington, IN, USA.

All participants were typically developing with no evidence of visual or developmental disorders that might impact performance. This was determined from recent clinic assessment records for the children, or by self-report for the adults. Children did not require optical correction and adults wore habitual corrective lenses (glasses or contact lenses) if needed.

Data from three 3-year-olds and one 5-year-old were excluded as a result of poor cooperation with the tasks. Data from a total of ten participants were analyzed in each age group in the main conditions, and from nine participants in the binocular control condition.

### Apparatus

Dichoptic stimulus presentation and the data collection were performed using MATLAB R2015a (The Mathworks, Inc) with Psychtoolbox version 3.0.12^48–50^ and the Eyelink Toolbox^51^. Gaze position was recorded using an Eyelink 1000 (SR Research, Ontario, Canada) sampling at 500 Hz, after performing a 9-point calibration for each participant. Stimuli were rear-projected from two vertically stacked Casio XJS 52 projectors (Casio, Shibuyu, Tokyo) using their green channels and circular polarization to stimulate the two eyes dichoptically. Participants wore the appropriate corresponding circular polarizers in a spectacle frame. The full projection screen covered 54 cm in width × 41 cm in height at a resolution of 1024 × 768 pixels. The data collected in Vancouver for the diplopia control condition were generated using liquid crystal shutter glasses (CrystalEyes 4) synchronized to a Viewsonic G225f. monitor with a refresh rate of 120 Hz. In both sets of equipment, there was no perceptible cross-talk between the images presented to the two eyes at the levels of luminance and contrast used here.

### Stimuli and procedure

Participants were stabilized at approximately 62 cm from the screen using a mounted chin rest, resulting in a pixel subtense of 3 arcmin. The stimuli for all tasks were cartoon images of four different randomly-ordered Pokémon characters, as used by Giaschi et al.^9,52^. They measured 2.2° horizontally by 2.9–3.1° vertically with a mean luminance of 182 cd/m^2^, and were presented in the center of the screen on a bright uniform background of 204 cd/m^2^.

#### Perceptual task

**Figure 5:**
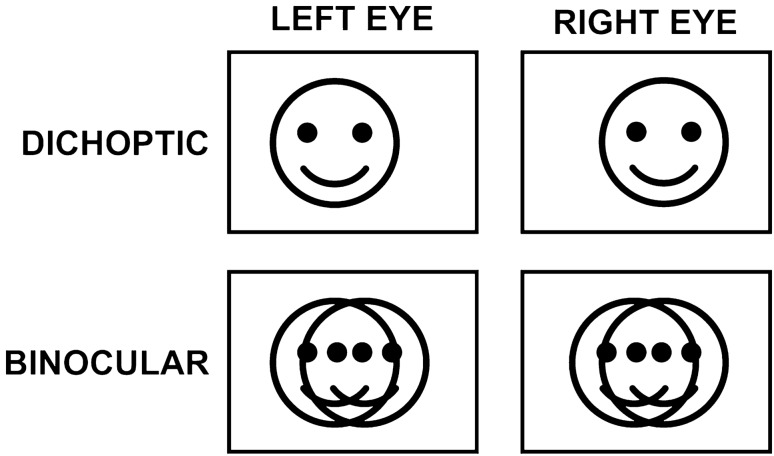
Top: Demonstration of a dichoptically presented disparity used in the diplopia threshold estimation task. The left eye image is slightly closer to the left side of the reference frame and the right eye image is slightly closer to the right side of the frame. This small disparity should result in the cartoon face appearing nearer than the reference frame if convergence is used to fuse the images. In this case, an observer should report seeing one face; if they are experiencing diplopia, they should report seeing two faces. Bottom: Example of a binocular stimulus used in the control condition. This stimulus has the same separation between the faces as the top row, but both faces are presented to both eyes. This stimulus should always lead to a report of seeing two characters. Note: the rectangular boxes used for illustration here were not included in the actual stimulus. The stimuli used in the experiment were green images of Pokemon characters, which have been replaced here with cartoon faces for copyright reasons.

The adults and 5-year-olds participated in a diplopia threshold assessment task^7^. Participants were instructed to watch the cartoon characters on the screen. At the beginning of each trial, a character was presented dichoptically for 1.5 s in the plane of the screen, with no interocular disparity (zero disparity). The character was then stepped to a different disparity for 320 ms before being replaced by a blank uniform luminance screen. This exposure duration was chosen to mirror previous work with these stimuli^9^. While this duration is long enough to initiate a reflexive vergence response (which occurs after approximately 160 ms in adults^30^), it limits the closed-loop corrective eye movements that can begin after approximately 320 ms. The trials consisted of steps to 0, 0.5, 1, 2, 4, or 8° of crossed (negative/near) and uncrossed (positive/far) test disparity, presented in a random order. Four trials were presented at each disparity, for a total of 44 trials. The trials were self-paced and after each disparity step participants were asked to report whether they perceived one or two characters, with an instruction to report ‘two’ if they perceived overlapping characters. Prior to undertaking this task, participants were given three practice trials to confirm they understood the task demands. The additional group of children was recruited to perform a control condition to confirm that this age group could reliably distinguish between single, overlapping and diplopic stimuli (Fig. 5). This condition consisted of the perceptual task described above for all disparities plus a series of randomly interleaved trials during which both characters were presented to both eyes at zero disparity, but at the separations used in the dichoptic trials. If participants were able to recognize and correctly report the percept of overlapping targets, they should consistently report seeing two characters during the binocular zero-disparity, overlapping character trials. Eye movements were not recorded during these perceptual tasks.

#### Vergence task

The adults, 5-year-olds, and 3-year-olds participated in the vergence response task. Participants were instructed to watch the cartoon characters on the screen. At the beginning of each trial, a single character was presented dichoptically on the screen with no interocular disparity. After the participant had fixated on the character for 1500 ms (within a gaze-contingent window of 24° horizontally and vertically), the cartoon was stepped to one of the crossed or uncrossed tested disparities of 0, 1, 2, 4, or 8° (for 3-year-olds), plus 0.5 and 16° (for 5-year-olds and adults). A stimulus step of 16° from a starting alignment at a 62 cm viewing distance corresponds to a converged viewing distance of approximately 15 cm from the observer in one direction and diverged beyond parallel alignment in the other direction. After 320 ms at the test disparity, participants viewed a blank uniform luminance screen while the eye-tracker continued to record for another 680 ms. To help engage their attention, children were also shown a photograph of an animal for three seconds after every five trials.

Twenty trials per disparity were collected from each adult and 5-year-old participant (260 trials total) except for two 5-year-old children who completed 14 trials per disparity (182 trials total). Adults completed all of the trials in one block, with each sequential set of all the disparities presented in a randomized order. For the 5-year-olds, trials were broken into three blocks. The 3-year-olds each completed 5 trials per disparity in one block (45 trials total), with breaks as necessary. Five 3-year-olds were able to complete a second block for a total of 10 trials per disparity (90 trials total).

### Data analysis

For the perceptual task, a Weibull function was fit to each participant’s proportion of “saw two” reports as a function of disparity step size, after collapsing across disparity sign (near and far). The diplopia threshold was defined as the 63% “saw two” point on the fitted function^53^.

For the eye-movement task, the data collected using the eye-tracker were pre-processed using a low-pass filter with a cutoff frequency of 20 Hz. Blinks were also detected and removed automatically. Epochs of missing data (either from blinks or poor eye-tracker video image quality) were linearly interpolated between the last sample before and the first sample after the missing interval. Trials with more than one blink or a blink during the stimulus presentation were excluded from the analysis. In total, < 1% of all trials were excluded from adult participants; 5% of all trials were excluded from 5-year-old participants; and 12% of all trials were excluded from 3-year-old participants.

Vergence position was then determined by subtracting the horizontal position of the left eye from the horizontal position of the right eye, and the vergence responses were averaged within each disparity step size for each participant in preparation for subsequent analyses. A positive vergence position indicates divergence from the plane of the screen, while a negative vergence position indicates convergence relative to the screen. Similarly, negative disparities represent crossed (near) stimuli, and positive disparities represent uncrossed (far) disparities.

**Figure 6:**
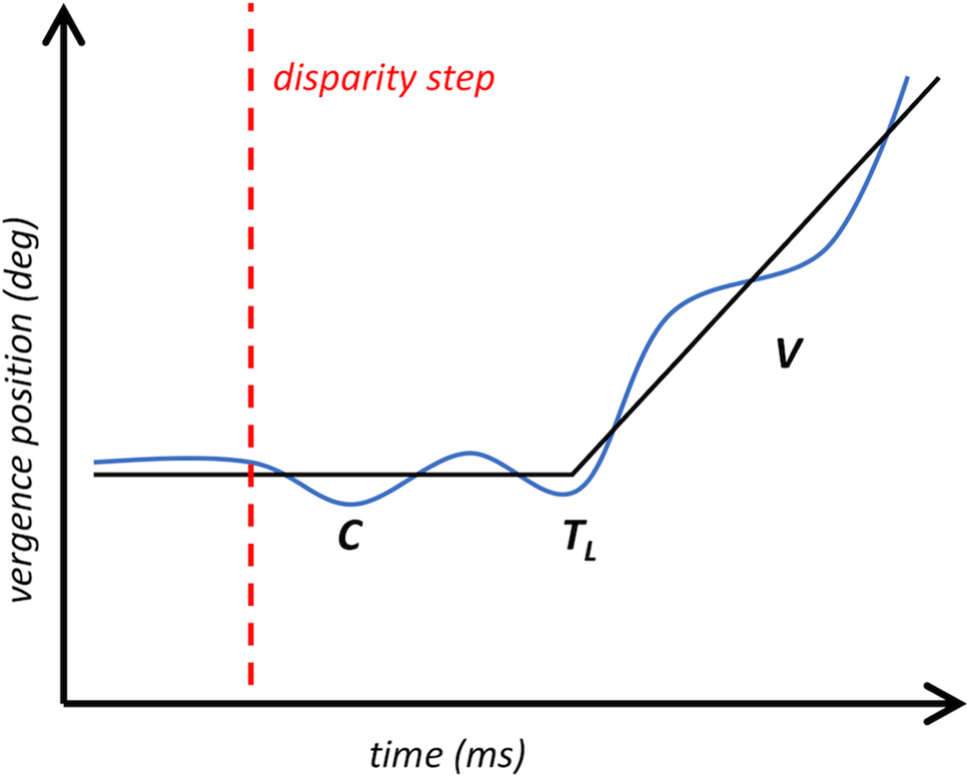
The fitting approach used to estimate the latency and velocity of vergence responses to disparity stimuli. C is a constant reflecting the stable vergence position during the initial latency period, T_L_ specifies one latency interval before the vergence position begins to change, and V is the slope of the line fit to the dynamic vergence response.

The velocity summary for the vergence response to a disparity step was quantified (in°/s) using the MATLAB *lsqnonlin* function. It was used to perform a least-squares error fit for a bilinear function of the form

1 $$ Y_{i} = C + (T_{i} > T_{L} )V(T_{i} > T_{L} ) $$

where Y = vergence position (in degree), C = the stable vergence position during the initial latency period, T_L_ = the latency (in ms) before the vergence position begins to change, and V = the slope of a line fit to the dynamic vergence response (Fig. 6). In a first iteration, this function was fit over a time window spanning from the onset of the disparity step to a manually-determined endpoint within a window of 400–600 ms later. The endpoint was selected to optimize the fit of the function to the data. The value of T_L_ obtained in this first stage was defined as one latency interval. A second, repeat fit to the data was then performed using a time window spanning from the onset of the disparity step to a period of two latency intervals later, with some shortening of the window when necessary to capture the underlying shape of the data. The value of V obtained from this second fit was used as the estimate of vergence response velocity (deg/s) for this participant to this disparity. Negative values indicate convergence toward the observer relative to the plane of fixation; positive values indicate divergence away from the plane of fixation. This approach was taken in an attempt to prevent visual feedback from modifying the velocity estimate; the velocity was estimated over any open-loop period of the response before a response adjustment could be initiated (e.g.^54,55^).

When the fit did not converge to establish an estimate of T_L_, or if the fit was influenced by a minor fluctuation leading to a latency estimate of less than 80 ms, the final fit was performed by taking the mean latency of the participant’s responses to the other disparities and calculating the slope of the vergence data spanning a window of between one and two of these latency intervals. This occurred, in particular, for the zero disparity stimuli or stimuli presented at very large disparities where there was a minimal vergence response.

## Data Availability

The datasets generated during and/or analysed during the current study are available from the corresponding author on reasonable request.
